# Childhood Asthma and Allergies in Urban, Semiurban, and Rural Residential Sectors in Chile

**DOI:** 10.1155/2013/937935

**Published:** 2013-05-23

**Authors:** Leonie Kausel, Anja Boneberger, Mario Calvo, Katja Radon

**Affiliations:** ^1^Biochemistry Faculty, Universidad Austral de Chile, Valdivia, Chile; ^2^Institute and Outpatient Clinic for Occupational, Social and Environmental Medicine, Hospital of the Ludwig-Maximilians University (LMU), 80336 Munich, Germany; ^3^Medical Faculty, Universidad Austral de Chile, Valdivia, Chile

## Abstract

While rural living protects from asthma and allergies in many countries, results are conflicting in Latin America. We studied the prevalence of asthma and asthma symptoms in children from urban, semiurban, and rural sectors in south Chile. A cross-sectional questionnaire study was conducted in semiurban and rural sectors in the province of Valdivia (*n* = 559) using the ISAAC (International Study of Asthma and Allergies in Childhood) questionnaire. Results were compared to prevalence in urban Valdivia (*n* = 3105) by using data from ISAAC III study. Odds ratios (+95% confidence intervals) were calculated. No statistical significant differences were found for asthma ever and eczema symptoms stratified by residential sector, but a gradient could be shown for current asthma and rhinoconjunctivitis symptoms with urban living having highest and rural living having lowest prevalence. Rural living was inversely associated in a statistical significant way with current asthma (OR: 0.4; 95% CI: 0.2–0.9) and rhinoconjunctivitis symptoms (OR: 0.3; 95% CI: 0.2–0.7) in logistic regression analyses. Rural living seems to protect from asthma and respiratory allergies also in Chile, a South American country facing epidemiological transition. These data would be improved by clinical studies of allergic symptoms observed in studied sectors.

## 1. Introduction

The prevalence of asthma has risen worldwide in the last few decades [1, 2] with Chile having a prevalence of childhood asthma of about 12% to 18% [3]. Countries like Chile, which currently experiences a stage of transition from low to high prevalence of atopic diseases in the population due to potential underlying environmental risk factors, offer the unique opportunity to validate the hypotheses that have been proposed in developed countries explaining the rising prevalence of atopic diseases, one of them stating that microbial exposure protects from atopic diseases (hygiene hypothesis) [4].

The hygiene hypothesis [5], states that modern health care and hygiene practices have led to a relative sterilization of the environment with reduced exposure to bacterial, viral, or fungal components. As a consequence, under these conditions, the proposed allergy-preventing potential of microbial factors is no more present in sufficient qualities and/or quantities [6]. In many studies, mainly from Europe, an inverse relationship between the degree of exposure to microbial pathogens in childhood and the development of disease was found [7–9].

In the same context, farming environment has been described to act as a protective factor in the development of allergic diseases and less consistently of asthma [10, 11]. Therefore, it has been suggested that people who grow up on a farm are exposed to certain immune modulatory factors that are specific to this lifestyle and protect against allergic diseases [12]. 

The hygiene hypothesis has not been widely studied in Chile [12, 13], yet evidence for the hygiene hypothesis is conflicting in Latin America [14]. The aim of the present study was to evaluate the hygiene hypothesis with regard to the development of asthma and allergies in children living in urban, semiurban, and rural residential sectors in the central south of Chile.

## 2. Methods

The International Study of Asthma and Allergies in Childhood (ISAAC) is a worldwide epidemiological research program established in 1991 to investigate asthma, rhinitis, and eczema in children [15]. Chile participated in two phases: in 1994 (phase I) and 2002 (phase III), respectively [1]. One of the Chilean centres of the ISAAC study was located in the city of Valdivia, capital of the Región de los Ríos, in the Central South of Chile (39°48′30′′ S latitude and 73°14′30′′ O longitude). Valdivia has an average annual relative humidity of 83% and an average temperature of 12°C. Main industries are forestry and agriculture. 

For the current study which was conducted in 2009, we decided to investigate the prevalence of asthma and allergies in public schools that were located in semiurban (2,000–100,000 inhabitants) and rural areas (<2,000 inhabitants) of the Región de los Ríos, so that we were able to compare these results with the ones obtained in the ISAAC study in urban city Valdivia (127,750 inhabitants). Residential sectors were determined only by population criterion. In all public schools in semiurban city Los Lagos (9,479 inhabitants) and three rural communities (Los Coihues, Folilco, and Nontuela) students in grades 6–8 (age range 10 to 16 years) were invited to participate in the questionnaire study. Children filled in the Chilean version of the ISAAC core questionnaire including questions about symptoms of asthma and allergies which had been used before in Valdivia [15, 16]. The questionnaire study was approved by the local ethic committee of the Universidad Austral de Chile (UACH) and had the support of the local representation of the Ministry of Education (Provincial de Educación). All students were free to participate in the study. 

Questionnaire data were double entered and error checked in an MS Access 2007 database. In order to compare the results of the present survey to the ISAAC III results in Valdivia, the data were restricted to the 13-14-year-old participants. ISAAC III results are only available for the 13-14-year-old children. Variables were defined and dichotomized (yes/no) as follows:

- ever smoked (Have you ever smoked in your life?);
- currently smoke (Do you smoke now?);
- asthma ever (Have you ever had asthma?);
- current asthma symptoms (Have you had wheezing or whistling in the chest in the last 12 months?);
- rhinoconjunctivitis symptoms (In the past 12 months, have you had a problem with sneezing, or a runny, or a blocked nose when you DID NOT have a cold or the flu? AND In the past 12 months, has this nose problem been accompanied by itchy-watery eyes?);
- eczema symptoms (“Have you ever had an itchy rash which was coming and going for at least 6 months? AND Has this itchy rash at any time affected any of the following places: the folds of the elbows, behind the knees, in front of the ankles, under the buttocks, or around the neck, ears, or eyes? AND Have you had these problems in the last 12 months?”).

### 2.1. Statistical Analyses

Cross-tabulation to visualize bivariate distributions of categorical predictors and outcomes was used. Logistic regression analyses adjusted for sex and current smoking [17] were performed. The data were analyzed with SPSS (PASW Statistics 17.0). All analyses were stratified by residential sector. 

## 3. Results

All students that were attending school the day the survey was made answered the questionnaire. About half of the population was female with less girls in rural schools (*P* = 0.08). Smoking was less frequently reported by students from semiurban schools than in urban or rural schools (*P* < 0.001) (Table 1).

**Figure 1:**
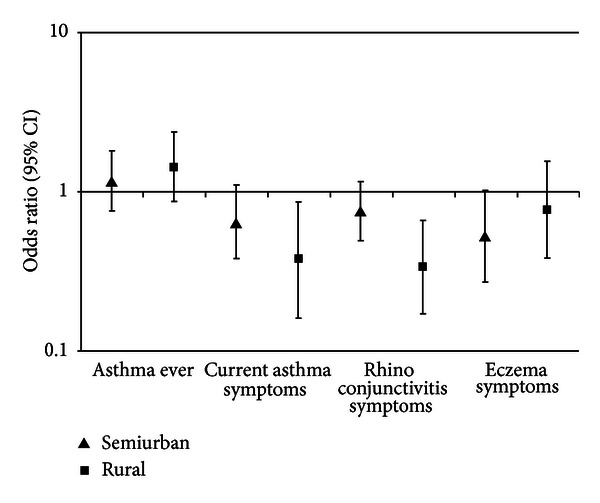
Odds ratio (adjusted for sex and current smoking) with 95% confidence interval by residential sector (Reference = Urban).

While asthma ever did not differ statistically significantly between residential sectors, a gradient could be shown for current asthma symptoms and rhinoconjunctivitis with highest prevalence in urban schools and lowest in rural schools (*P* < 0.001). Skin symptoms showed highest prevalence in urban schools, but was higher in rural than in semiurban schools, although the difference was not significant. These results were confirmed when adjusted for sex and current smoking (Table 2) and are shown in Figure 1.

## 4. Discussion

In our study, based on questionnaires, for the first time asthma and asthma-related symptoms prevalence were evaluated in urban, semiurban, and rural residential sectors in the central south of Chile. We could confirm a lower prevalence of current asthma symptoms and symptoms of rhinoconjunctivitis in rural schools as compared to semiurban and urban schools in the central south of Chile. These findings may support the hygiene hypothesis in a population currently in the stage of epidemiologic transition. However, they contradict findings from studies in inner-city homes in the USA [18]. Clinical studies might be helpful to clarify the observed prevalence in the analyzed Chilean sectors.

We included a relative high number of students from rural and semiurban schools. However, these numbers were smaller than the ones from urban Valdivia. One has to bear in mind that semiurban and especially rural areas in the central south of Chile are frequently hard to reach and only a low number of students is found per school. As our results indicated statistical significant differences, the power of our study was sufficient to show the hypothesized effect.

Response was high so that no major selection bias could be anticipated. As potential confounders, we only included sex and current smoking. Age range was restricted to only 13-14 years old, so that confounding by age seems to be unlikely. In addition, socioeconomic status could be a potential confounder [19]. However, as we only included public schools the socioeconomic status is expected to be low throughout the study population. 

One disadvantage is that the study in the rural and semiurban schools was done seven years after the ISAAC study in Valdivia. As resources were limited, the ISAAC study could unfortunately not be repeated at the time of this study. If the prevalence of symptoms had changed over the past seven years in urban Valdivia, it most likely increased. Therefore, the differences shown in this study might underestimate the true difference. 

The reason for the markedly lower current smoking prevalence in semiurban schools is hard to explain. Chance seems to be the most likely explanation. In general, current smoking in adolescents seems high in Chile [20]. Unfortunately, no data on environmental tobacco smoke exposure especially in early years were available. A study from Germany showed that current smoking is more prevalent in urban than in rural areas [21], a fact that seems plausible for Chile, too. This might be one factor that may explain part of the observed difference in asthma prevalence. However, it is not expected to influence the lower prevalence of rhinoconjunctivitis in rural areas. 

It is important to recall that urban area is the major city of Valdivia (127,750 inhabitants), semiurban area is the city of Los Lagos (9,479 inhabitants), and rural area is in the middle of pre-Cordilleran Chilean landscape in the province of Valdivia, Chile (<1000 inhabitants). The environmental conditions of the sectors are truly different when considering exposure to allergens such as dust, pollen, and animals. The results obtained in this study are concordant with studies carried out in Europe, USA, and New Zealand, which show that farmer environments have a protective effect regarding the development of atopic diseases [10, 22, 23]. 

In conclusion, a marked protective effect of rural environment on asthma and rhinoconjunctivitis symptoms could be seen. The observed phenomenon supports the compliance of the hygiene hypothesis for the central south of Chile. It would be of great interest to continue investigating this phenomenon with a bigger study population from semiurban and rural areas.

## Figures and Tables

**Table 1:** Description and symptoms prevalence stratified by residential sector.

	Total (*N* = 3363)	Residential sector			
		Urban (*n* = 3105)*	Semiurban (*n* = 159)*	Rural (*n* = 100)*	*P* chi^2^
Female	3363	1687 (54.3%)	86 (54.1%)	43 (43.0%)	0.08
Ever smoked	3356	1659 (53.4%)	36 (22.6%)	49 (59.0%)	<0.0001
Current smoker	3339	501 (16.2%)	7 (4.4%)	14 (14.0%)	0.001
Asthma ever	3354	490 (15.8%)	28 (17.6%)	20 (20.0%)	0.32
Current asthma symptoms	3344	496 (16.0%)	16 (10.1%)	6 (6.0%)	0.008
Rhinoconjunctivitis symptoms	3343	816 (26.3%)	28 (17.6%)	10 (10.0%)	<0.001
Eczema symptoms	3336	506 (16.3%)	10 (6.3%)	9 (9.0%)	0.04

*n  is restricted to the 13-14-year-old participants.

**Table 2:** Association between residential sector and asthma and associated symptoms stratified by residential sector in the central south of Chile.

Symptoms	OR (adjusted for sex and current smoking) (95% CI)		
	Urban (reference)	Semiurban	Rural
Asthma ever (*n* = 3347)	1	1.2 (0.8–1.8)	1.4 (0.9–2.4)
Current asthma symptoms (*n* = 3338)	1	0.6 (0.4–1.1)	0.4 (0.2–0.9)
Rhinoconjunctivitis symptoms (*n* = 3338)	1	0.8 (0.5–1.2)	0.3 (0.2–0.7)
Eczema symptoms (*n* = 3332)	1	0.5 (0.3–1.0)	0.8 (0.4–1.6)
